# Gene delivery of hypoxia-inducible VEGF targeting collagen effectively improves cardiac function after myocardial infarction

**DOI:** 10.1038/s41598-017-13547-1

**Published:** 2017-10-16

**Authors:** Jing-Bo Xia, Hai-Yan Wu, Bing-Lin Lai, Li Zheng, Deng-Cheng Zhou, Zao-Shang Chang, Cheng-Zhou Mao, Guang-Hui Liu, Kyu-Sang Park, Hui Zhao, Soo-Ki Kim, Guo-Hua Song, Dong-Qing Cai, Xu-Feng Qi

**Affiliations:** 1 Key Laboratory of Regenerative Medicine of Ministry of Education, Department of Developmental & Regenerative Biology, Jinan University, Guangzhou, 510632 China; 2 College of Environmental Science and Engineering, Guangdong University of Technology, Guangzhou, 510006 China; 3 Department of Physiology, Wonju College of Medicine, Yonsei University, Wonju, Gangwon 220-701 Korea; 4 Key Laboratory of Regenerative Medicine of Ministry of Education, School of Biomedical Sciences, Faculty of Medicine, The Chinese University of Hong Kong, Hong Kong SAR, China; 5 Department of Microbiology, Yonsei University Wonju College of Medicine, Wonju, Gangwon 220-701 Korea; 6 Institute of Atherosclerosis, TaiShan Medical University, Tai’an, 271000 China

## Abstract

Vascular endothelial growth factor (VEGF) plays important roles in improvement of cardiac function following myocardial infarction (MI). However, the lack of a steerable delivery system of VEGF targeting the infarcted myocardium reduces the therapeutic efficacy and safety. Here, we constructed a series of lentiviral vector systems which could express a fusion protein consisted of a collagen-binding domain (CBD) and hVEGF (CBDhVEGF), under the control of 5HRE-hCMVmp (5HRE), the hypoxia-inducible promoter consists of five copies of the hypoxia-responsive element (HRE) and a human cytomegalovirus minimal promoter (hCMVmp). We demonstrated that 5HRE has the comparable ability to strongly drive CBDhVEGF under hypoxic condition as the ubiquitous CMV promoter, but it can hardly drive target gene under normoxic condition. 5HRE-drived CBDhVEGF specifically bound to type I collagen and significantly promoted the viability of HUVEC cells. Moreover, after injection of lentivirus into heart of mouse with MI, CBDhVEGF was mainly retained in infarcted myocardium where containing rich collagen and significantly improved angiogenesis and cardiac function when compared with hVEGF. Moreover, CBDhVEGF mediated by lentivirus has little leakage from infarcted zone into blood than hVEGF. Taken together, our results indicate that 5HRE-CBDhVEGF lentiviral vector system could improve cardiac function in the collagen-targeting and hypoxia-inducible manners.

## Introduction

The leading cause of death in the world is coronary heart disease (CHD) which is usually caused by coronary stenosis, thereby leading to ischemic and hypoxic injury myocardium, also known as myocardial infarction (MI). Vascular endothelial growth factor (VEGF) is a major regulator of blood vessel formation through promoting endothelial cells (ECs) proliferation, migration and survival^1^. It has been reported that the majority of ECs within vasculature remain quiescent during adulthood and proliferate only after angiogenic activation mostly by stimulation of VEGF^1,2^. Therefore, VEGF-mediated angiogenesis is integral for tissue restoration in cases of injury, ischemia and wound healing^3^. Many studies have demonstrated that the administration of recombinant VEGF protein or VEGF gene into ischemic myocardium has been shown to enhance collateral vessel flow and improve cardiac function^4–6^. Although the beneficial effects of VEGF have been suggested in previous studies, its safety is still a major concern. High doses of VEGF can lead to pathologic disease manifestation including atherosclerosis and hemangioma formation^3,7^. Therefore, a delicate balance should exist between the therapeutic benefits of VEGF and its deleterious repercussions^8^.

The cardiac extracellular matrix (ECM) plays an important role in tissues support, cell survival and proliferation^9^. The type I (about 80%) and type III (about 10%) collagen are the main components of cardiac ECM^9–11^. It has been shown that the production of type I collagen increases in ischemic area after MI^12,13^. Therefore, type I collagen may be used as the target for certain growth factors to improve cardiac function. Indeed, fusion proteins of VEGF or SDF-1α combined with a polypeptide TKKTLRT named collagen-binding domain (CBD) have been demonstrated to significantly improve cardiac function after MI^5,14,15^. However, the rapid biodegradation and relatively short biological half-life of VEGF are the major limitations in delivering such a protein^16^. Thus, a stable and steerable delivery system for VEGF targeting the injured myocardium may offer an optimal therapy for MI.

Hypoxia is one of unique features of MI and has been considered as a major factor for targeting therapy. A hypoxia-responsive promoter, 5HRE-hCMVmp consisting of five copies of a 35-bp fragment from the hypoxia-responsive element (HRE) of the human VEGF gene and a human cytomegalovirus minimal promoter (hCMVmp), has been reported previously^17,18^. Moreover, it has been demonstrated that HREs combined with a minimal simian virus 40 promoter or with a minimal MLC-2v promoter could specifically drive VEGF expression in ischemic mouse heart^19,20^. The previous studies indicate that HREs may be a suitable element to drive target gene expression under hypoxic conditions via combination with minimal ubiquitous or tissue specific promoters.

In the present study, we designed a series of lentiviral vector systems which can express hVEGF alone or a fusion protein consisting of the collagen-binding domain and hVEGF (CBDhVEGF) under the control of 5HRE-hCMVmp (5HRE) or the ubiquitous CMV promoter (see Methods and Figure S1). We demonstrated that the lentiviral vectors-expressed CBDhVEGF could specifically bind to type I collagen and maintain the biological activity similar with hVEGF *in vitro*. In the mice acute myocardial infarction (AMI) model, we found that the CBDhVEGF driven by 5HRE could be expressed and retained in the ischemic and hypoxic myocardium, thereby improving the cardiac function by increasing the arteriole and capillary density, with the comparable capacity of that driven by the ubiquitous CMV promoter.

## Results

### Hypoxia-responsive ability of 5HRE promoter

**Figure 1:**
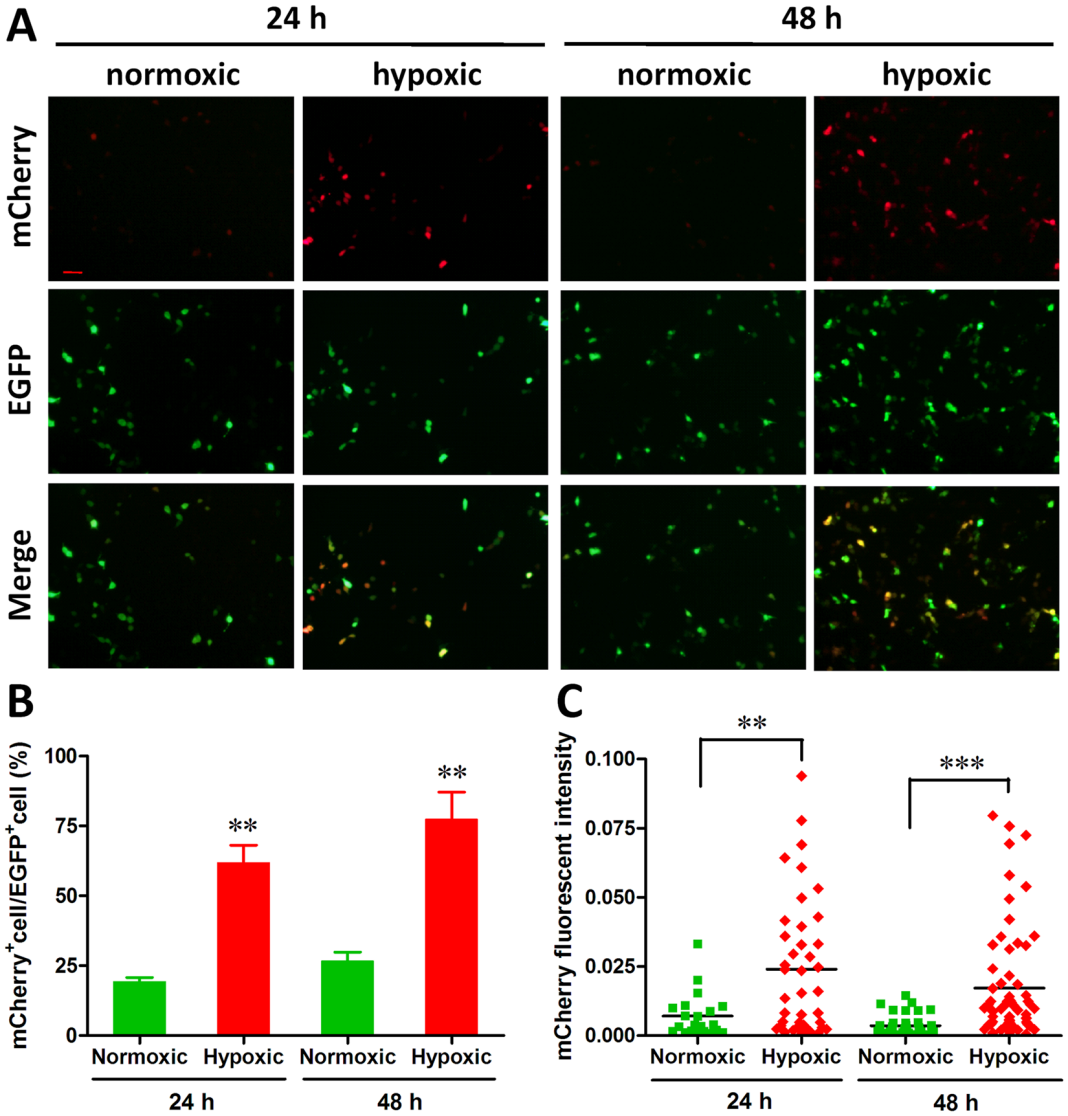
*In vitro* hypoxia-responsive ability of 5HRE-hCMVmp promoter. HEK293T cells transfected with pLOX5HRE-mCherry-E/P vector were incubated under normoxic or hypoxic conditions for 24–48 h, respectively. The expressions of mCherry and EGFP were examined by fluorescence microscopy. (**A**) A representative microscopic image for each condition is shown (scale bar = 50 μm). (**B**) Percentage ratio of mCherry positive cells to EGFP positive cells was examined as described in Methods section. **p < 0.01. (**C**) The quantification of mCherry fluorescence intensity was performed as described in Methods section. **p < 0.01, ***p < 0.001.

Firstly, the responsive ability of 5HRE-hCMVmp promoter to hypoxia was evaluated using HEK293T cells *in vitro*. As shown in Fig. 1A, similar expression levels of EGFP driven by EF1α promoter were observed 24 h after transfection with pLOX5HRE-mCherry-E/P vectors under normoxic and hypoxic conditions. However, strong expression of mCherry driven by 5HRE-hCMVmp promoter was detected under hypoxic condition, but not under normoxic condition. Similar results were also observed in HEK293T cells transfected with pLOX5HRE-mCherry-E/P vectors for 48 h. Statistic analysis showed that the percentage ratio of mCherry^+^ cells to EGFP^+^ cells were significantly increased by hypoxic treatment for 24 h and 48 h, respectively (Fig. 1B). Moreover, markedly increases in the mCherry fluorescence intensity were also observed in cells treated with hypoxia (Fig. 1C).

### Controlled expressions of hVEGF and CBDhVEGF under hypoxic conditions

**Figure 2:**
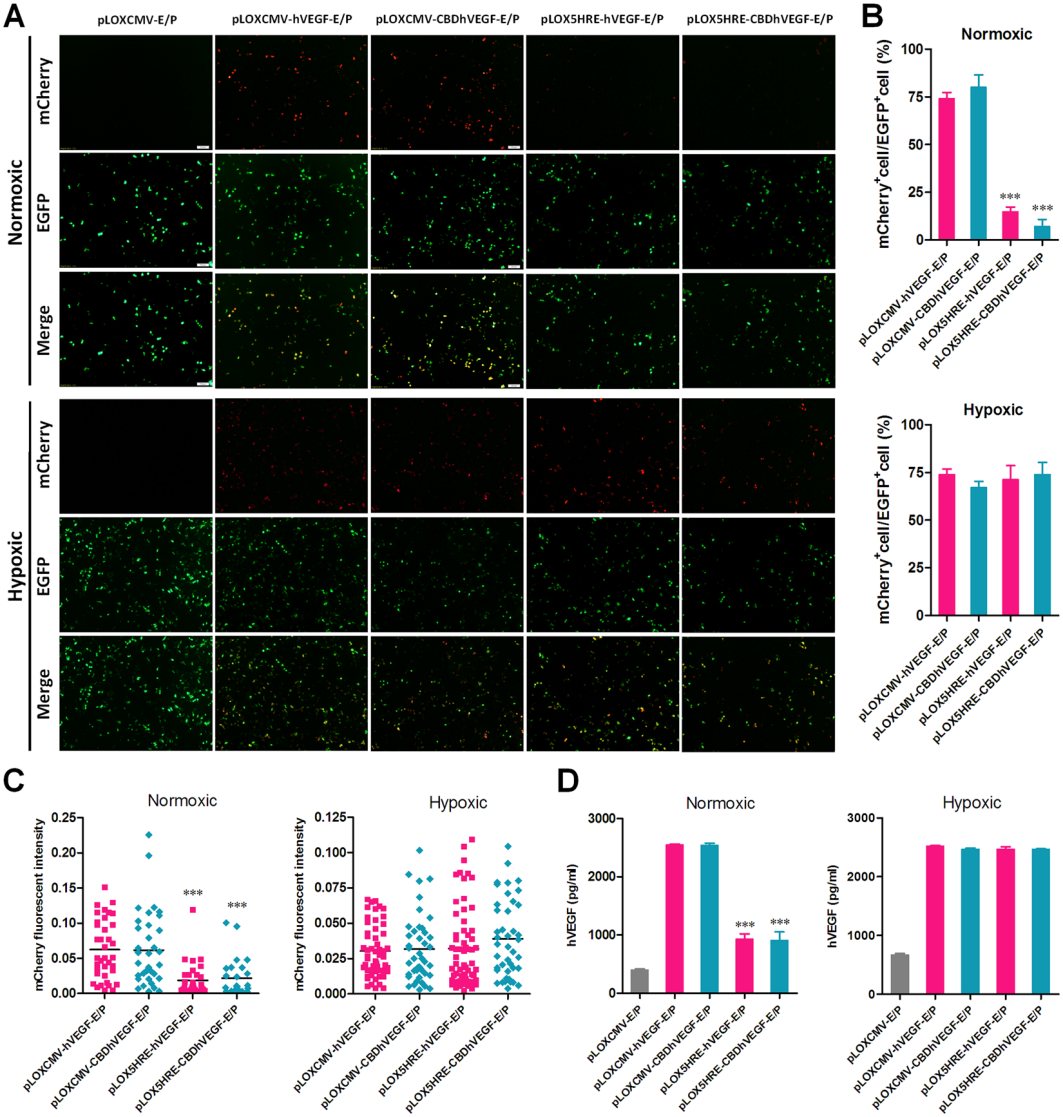
Controlled expressions of hVEGF-Flag-T2A-mCherry and CBD-hVEGF-Flag-T2A-mCherry fusion proteins under hypoxic conditions. HEK293T cells were transfected with various pLOXCMV-EP-based lentiviral vectors and incubated under normoxic or hypoxic conditions for 24 h. (**A–C**) The expression of mCherry and EGFP was examined by fluorescence microscopy. (**A**) A representative microscopic image for each condition is shown (scale bar = 100 μm). (**B**) Percentage ratio of mCherry positive cells to EGFP positive cells was examined as described in *SI Materials and Methods*. ***p < 0.001 versus CMV promoter. (**C**) The quantification of mCherry fluorescence intensity was performed as described in *SI Materials and Methods*. ***p < 0.001 versus CMV promoter. (**D**) VEGF concentration in the culture supernatants were analyzed by a human VEGF ELISA Kit. ***p < 0.001 versus CMV promoter.

To further test the expression of hVEGF-2A-mCherry and CBDhVEGF-2A-mCherry fusion proteins, a series of pLOX-E/P-based vectors were transfected into HEK293T cells for 24 h. As shown in Fig. 2A, strong expressions of EGFP were detected in each group treated with pLOXCMV-E/P, pLOXCMV-hVEGF-E/P, pLOXCMV-CBDhVEGF-E/P, pLOX5HRE-hVEGF-E/P, or pLOX5HRE-CBDhVEGF-E/P vectors, in despite of normoxic or hypoxic conditions. Under normoxic conditions, robust expression of mCherry was observed in cells treated with pLOXCMV-hVEGF-E/P or pLOXCMV-CBDhVEGF-E/P vectors. However, the red fluorescence of mCherry was almost undetectable in cells treated with pLOX5HRE-hVEGF-E/P or pLOX5HRE-CBDhVEGF-E/P vectors. Except pLOXCMV-E/P, the empty vector, the expressions of mCherry were observed in all groups treated by the other four vectors under hypoxic conditions. Statistic analysis showed that the percentages of EGFP^+^/mCherry^+^ double positive cells in groups treated with pLOX5HRE-hVEGF-E/P or pLOX5HRE-CBDhVEGF-E/P vectors were significantly lower than that in groups treated with pLOXCMV-hVEGF-E/P or pLOXCMV-CBDhVEGF-E/P vectors under normoxic conditions. But the levels of EGFP^+^/mCherry^+^ cells were similar under hypoxic conditions (Fig. 2B). Furthermore, the fluorescent intensity of mCherry also showed the similar change like the EGFP^+^/mCherry^+^ cell percentage (Fig. 2C). In addition, the productions of VEGF expressed by pLOX5HRE-hVEGF-E/P or pLOX5HRE-CBDhVEGF-E/P vectors were significantly lower compared with that expressed by pLOXCMV-hVEGF-E/P or pLOXCMV-CBDhVEGF-E/P vectors under normoxic conditions. But the productions of VEGF mediated by 5HRE promoter reached a high level like that mediated by CMV promoter under hypoxic conditions (Fig. 2D). These findings suggest that 5HRE promoter is hypoxia-responsive and that it can control the expression of target gene depending on hypoxia.

### Both hVEGF and CBDhVEGF expressed by pLOX5HRE-based vectors promote HUVEC viability

**Figure 3:**
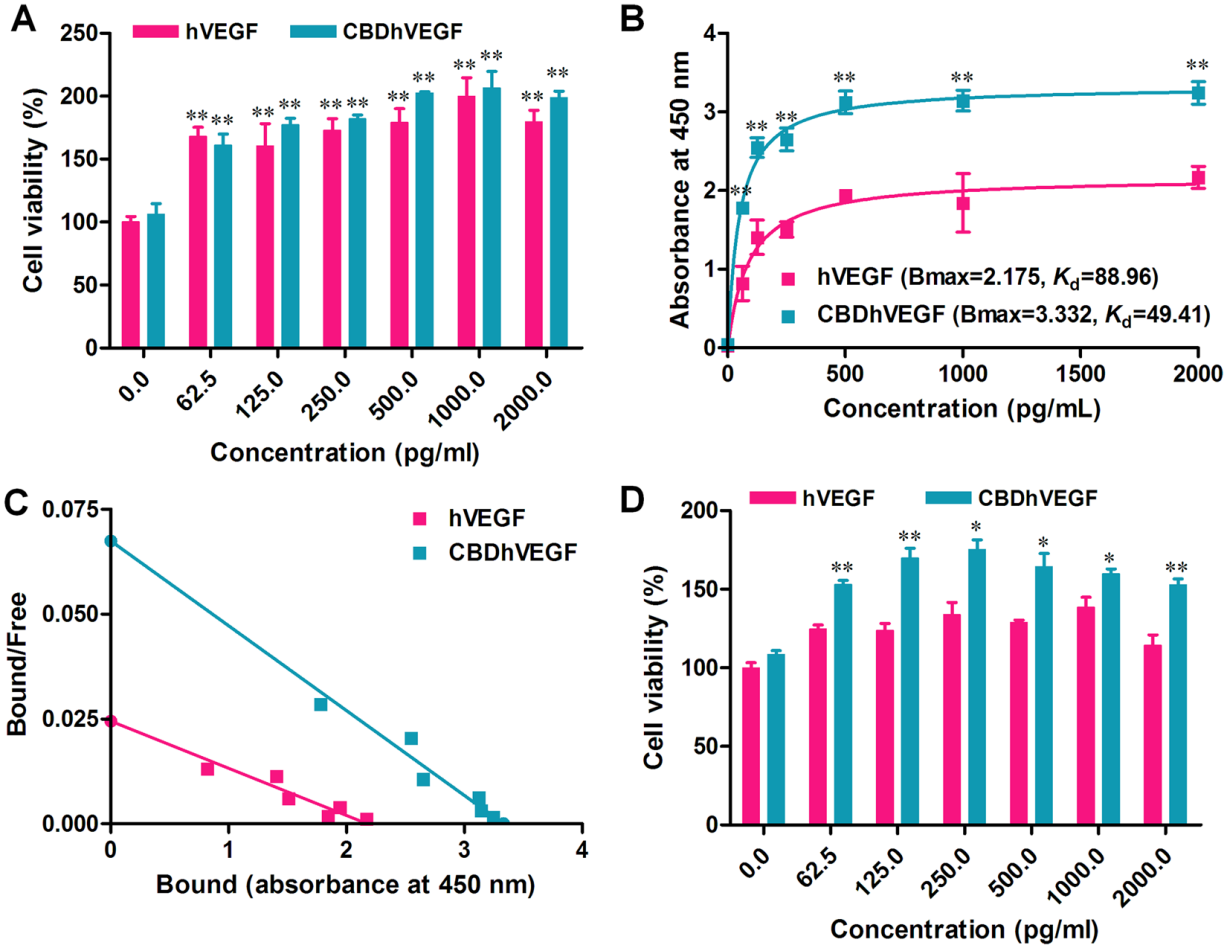
Biological activity and binding ability of CBDhVEGF to collagen. (**A**) HUVECs were incubated with hVEGF or CBDhVEGF (0–2,000 pg/mL, n = 4 for each dose) for 48 h. The cell growth-promoting activity was examined by CC-K8 Cell Counting Kit and expressed as the percentage of cell viability of resting cells. Results are presented as mean ± SEM of three separate experiments conducted in duplicate, **p < 0.01 versus control group. (**B**) The amount of hVEGF or CBDhVEGF bound to collagen was measured by ELISA assay (n = 4 for each dose). Results are presented as mean ± SEM of three separate experiments conducted in duplicate, **p < 0.01 versus hVEGF. (**C**) The *K*_d_ values for binding of hVEGF or CBDhVEGF to collagen were calculated by Scatchard analysis. Slope of each line = −1/*K*_d_. (D Collagen-coated plates were washed extensively after incubation with hVEGF or CBDhVEGF. The effects of retained hVEGF or CBDhVEGF in collagen on HUVECs growth were determined by cell counting assay (n = 4 for each dose). Results are presented as mean ± SEM of three separate experiments conducted in duplicate, *p < 0.05, **p < 0.01 versus hVEGF.

The typical biological activity of VEGF is to promote the viability of endothelial cells. We next examined whether hVEGF or CBDhVEGF expressed by pLOX5HRE-based vectors under hypoxic condition retained their biological activity to promote HUVECs viability. As shown in Fig. 3A, both hVEGF and CBDhVEGF mediated by pLOX5HRE-based vectors in HEK293T cells under hypoxic conditions could significantly promote the viability of HUVECs in a concentration-dependent manner. Thus, 5HRE-mediated VEGF still retains its biological activity. Furthermore, there was no significant difference between hVEGF group and CBDhVEGF group, which indicated that the biological activity of VEGF was not affected by its fusion with the CBD peptides at the N-terminus.

### *in vitro* binding kinetics and affinity of CBDhVEGF expressed by pLOX5HRE-based vector

To evaluate the *in vitro* collagen-binding activities of hVEGF and CBDhVEGF produced by pLOX5HRE-based vectors, growth factors binding to type I collagen were measured by a modified ELISA assay. As shown in Fig. 3B, the binding curves of hVEGF and CBDhVEGF were significantly different. The absorbance at 450 nm in CBDhVEGF was significantly higher than that of hVEGF at a concentration range from 62.5 to 2000 pg/mL (*p* < 0.01 respectively), which indicates that more CBDhVEGF bound to type I collagen than hVEGF. The binding curves were used to calculate the *K*_d_ values of hVEGF or CBDhVEGF binding to collagen (3 μg) by Scatchard analysis and the slope of the resulting straight line equals -/*K*_d_. The *K*_d_ value of hVEGF or CBDhVEGF binding to type I collagen was calculated as 88.96 or 49.41 pg/mL, respectively. The lower *K*_d_ value of CBDhVEGF represented a higher affinity to collagen, which indicates that CBDhVEGF binds more specifically to collagen that hVEGF (Fig. 3C). Moreover, the biological activity of CBDhVEGF binding to collagen was verified by HUVECs viability analysis. As shown in Fig. 3D, both hVEGF and CBDhVEGF binding to collagen could promote the viability of HUVECs in a concentration dependent manner. However, HUVECs seeded in CBDhVEGF-binding collagen showed significantly higher viability ability than that in hVEGF-binding collagen, which indicated that CBDhVEGF has higher affinity to collagen that hVEGF.

### Lentivirus-mediated CBDhVEGF improves cardiac function after myocardial infarction

**Figure 4:**
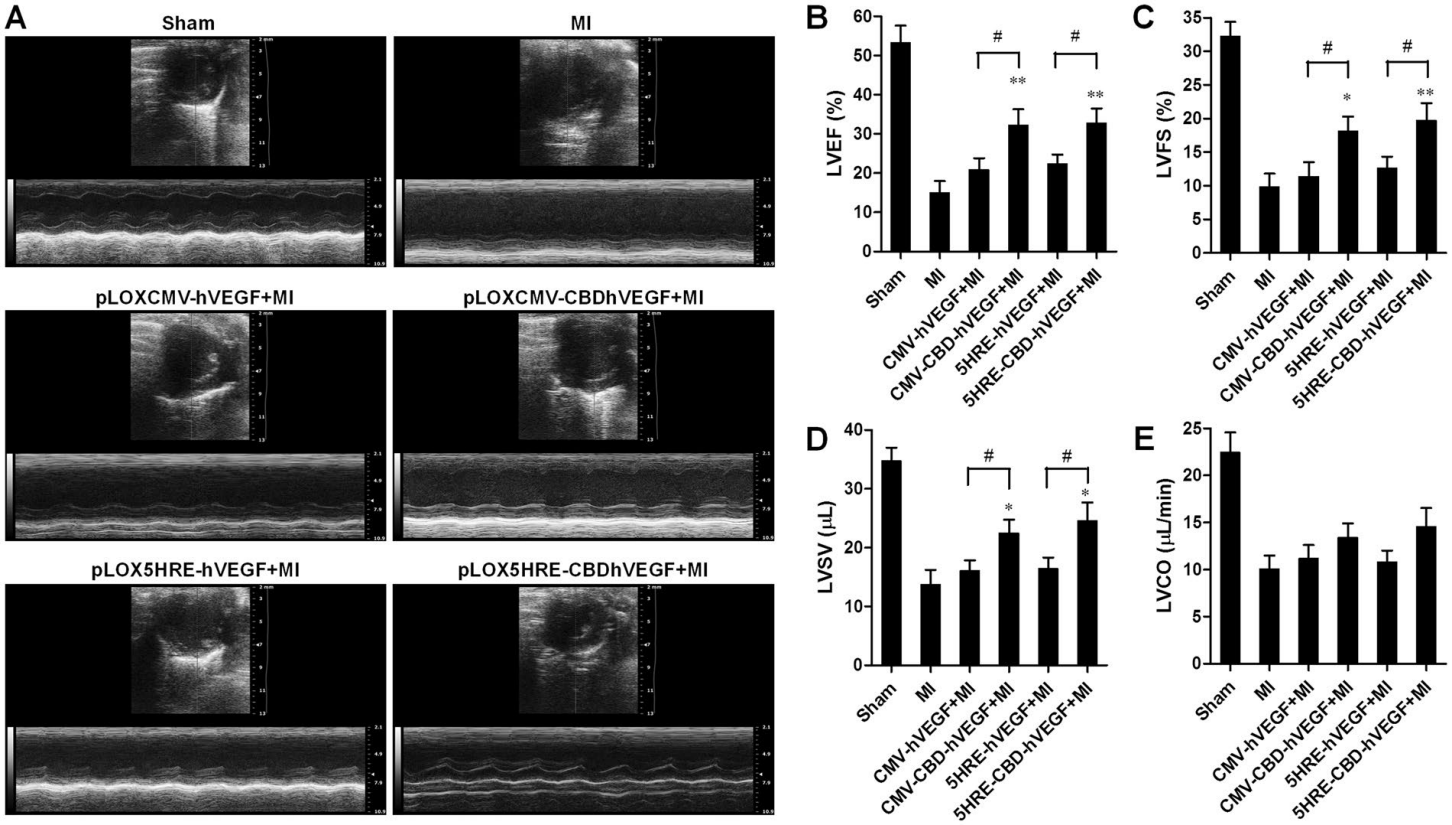
Evaluation of left ventricle function by echocardiography. (**A**) Representative images of M-mode echocardiography showing improved cardiac function in mice treated with lentivirus expressing hVEGF or CBDhVEGF compared with mice treated with control virus. (**B–E**) The left ventricular ejection fraction (LVEF), fractional shortening (LVFS), stroke volume (LVSV), and cardiac output (CO) were calculated by M-mode echocardiography in each group. Values are presented as mean ± SEM (n = 8 for each group). *p < 0.05, **p < 0.01, ^#^ *p* < 0.05.

To evaluate the effects of hVEGF or CBDhVEGF on cardiac function, lentiviruses based on pLOXCMV-hVEGF, pLOXCMV-CBDhVEGF, pLOX5HRE-hVEGF, or pLOX5HRE-CBDhVEGF vectors were injected into 3 sites in the infarct zone after LAD. Cardiac function was evaluated by echocardiography at 10 days after MI. As shown in Fig. 4A, MI-induced decreases in left ventricular (LV) systolic function were improved in various degrees by the injection of lentiviruses. The LV ejection fraction (LVEF) was significantly increased in groups treated with lentiviruses expressing hVEGF or CBDhVEGF driven by CMV or 5HRE promoters compared with MI group. However, CBDhVEGF driven by CMV or 5HRE promoters significantly increased the LVEF values when compared with hVEGF (Fig. 4B). In addition, both LV fractional shortening (LVFS) and LV stroke volume (LVSV) were significantly higher in CMV- or 5HRE-driving CBDhVEGF groups than that in hVEGF groups, respectively (Fig. 4C and D). Importantly, 5HRE-driving CBDhVEGF has comparable ability to improve cardiac function compared to that driven by CMV promoter. No significant differences were observed in cardiac output (CO) among the control (MI), hVEGF and CBDhVEGF groups (Fig. 4E).

To evaluate the morphology and fibrosis of myocardium, H&E and Masson trichrome staining were performed. As shown in Figure S2A, no cardiomyocytes with integral morphology were observed in the infarct zone in control group. However, experimental groups injected with lentivirus expressing hVEGF or CBDhVEGF showed some cardiomyocytes with Integral morphology. Furthermore, CBDhVEGF mediated by lentiviruses could greatly decrease the Masson trichrome positive area in infarct zone compared with hVEGF or control groups, which indicates that CBDhVEGF is capable of protecting myocardium against fibrosis (Figure S2B–D). Taken together, these results demonstrated that CBDhVEGF mediated by lentiviruses improved cardiac function and myocardium morphology after MI.

To further confirm the controlled expression of CBDhVEGF driven by 5HRE promoter *in vivo*, lentivirus including pLOXCMV-CBDhVEGF or pLOX5HRE-CBDhVEGF were injected into hearts of mice with or without MI treatment for 6 weeks, heart samples were then analyzed in different time points. As shown in Figure S3, little expression of report gene driven by CMV promoter was observed 3 days after virus injection in mice with or without MI treatment. The expression of report gene reached a peak at day 14 and maintained up to 42 days. For pLOX5HRE-CBDhVEGF lentivirus, robust signals of report gene were detected in the infarcted myocardium in the manner of pLOXCMV-CBDhVEGF lentivirus. However, in the sham-operated group (without MI), there was no fluorescent signals were detected in the cardiac area with injection of pLOX5HRE-CBDhVEGF virus from 3 to 42 days. Importantly, it should be noted that continuous expression of VEGF driven by CMV promoter indeed results in abnormal angiogenesis (Figure S4), as previously reported^3,7^. These findings suggest that 5HRE-CBDhVEGF system is inducible upon hypoxia and safer to therapy MI *in vivo*, compared with CMV promoter.

### Hypoxia-inducible CBDhVEGF binds to infarct myocardium

To examine the *in vivo* responsibility of 5HRE promoter to hypoxia, the expression of mCherry, the reporter gene fused with the C-terminuses of hVEGF-Flag or CBDhVEGF-Flag through a T2A peptide, was firstly examined by fluorescence microscopy. As shown in Figure S5A, no red fluorescence signaling was detected in control group injected with lentivirus based on pLOXCMV vector. However, robust expression of mCherry was observed in experimental groups injected with lentivirus based on pLOXCMV-hVEGF, pLOXCMV-CBDhVEGF, pLOX5HRE-hVEGF, or pLOX5HRE-CBDhVEGF vectors. Statistic analysis showed that both the fluorescence intensity of mCherry and the percentage of mCherry positive cells were significantly higher in 5HRE-based lentivirus groups than that in CMV-based lentivirus groups, respectively (Figure S5B and C). These findings suggest that 5HRE promoter is powerful to drive target gene *in vivo*.

**Figure 5:**
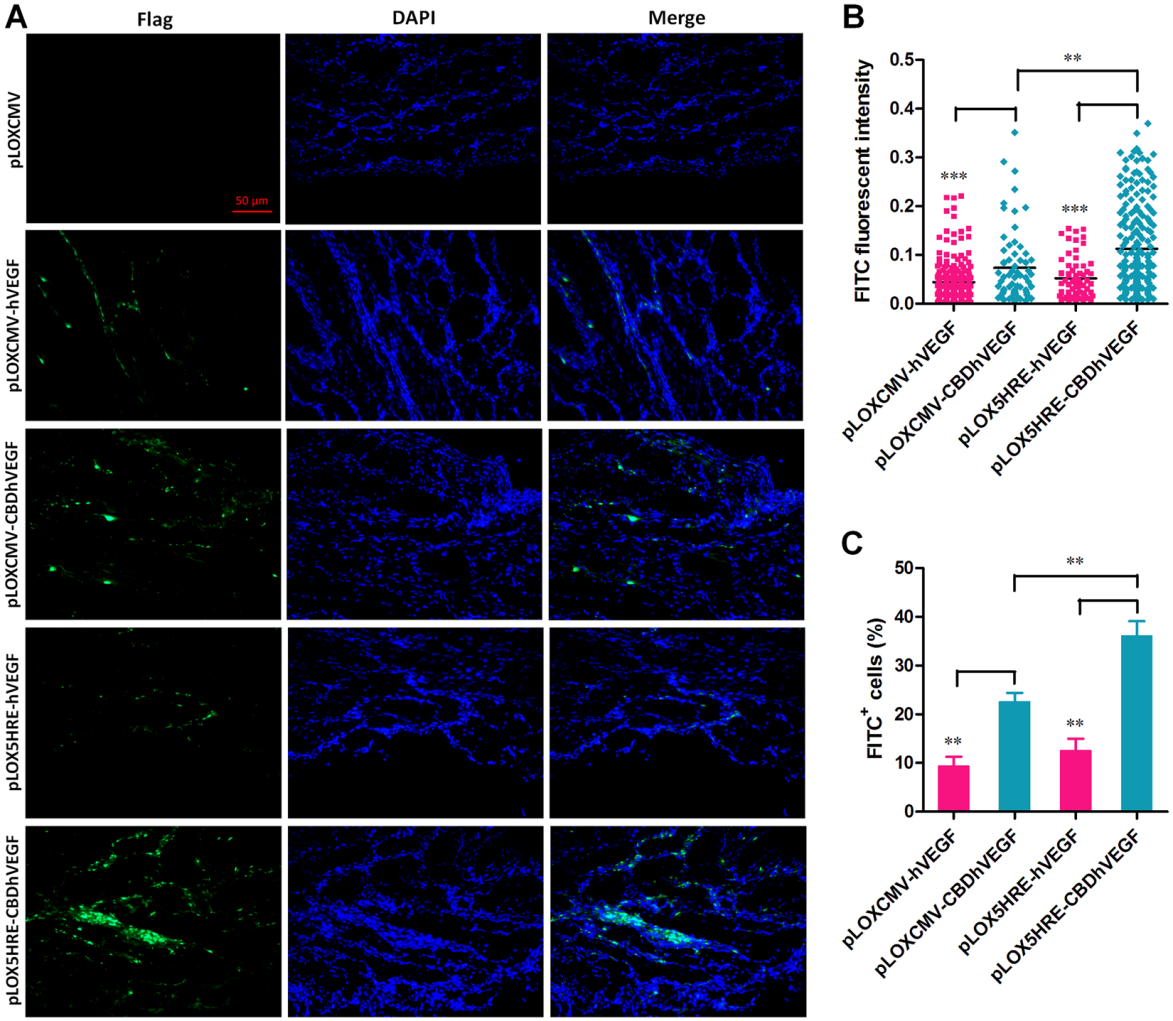
Expression of Flag tag in hypoxic myocardium. The expression of Flag tag, which was fused with C-terminus of VEGF, was detected in ischemic myocardium by immunofluorescence staining. (**A**) Representative images for each condition is shown (scale bar = 50 μm). (**B**) The quantification of FITC fluorescence intensity was performed as described in Methods section. Results are presented as mean ± SEM (n = 8), **p < 0.01, ***p < 0.001. (**C**) Percentage ratio of FITC positive cells was examined as described in Methods section. Results are presented as mean ± SEM (n = 8), **p < 0.01.

**Figure 6:**
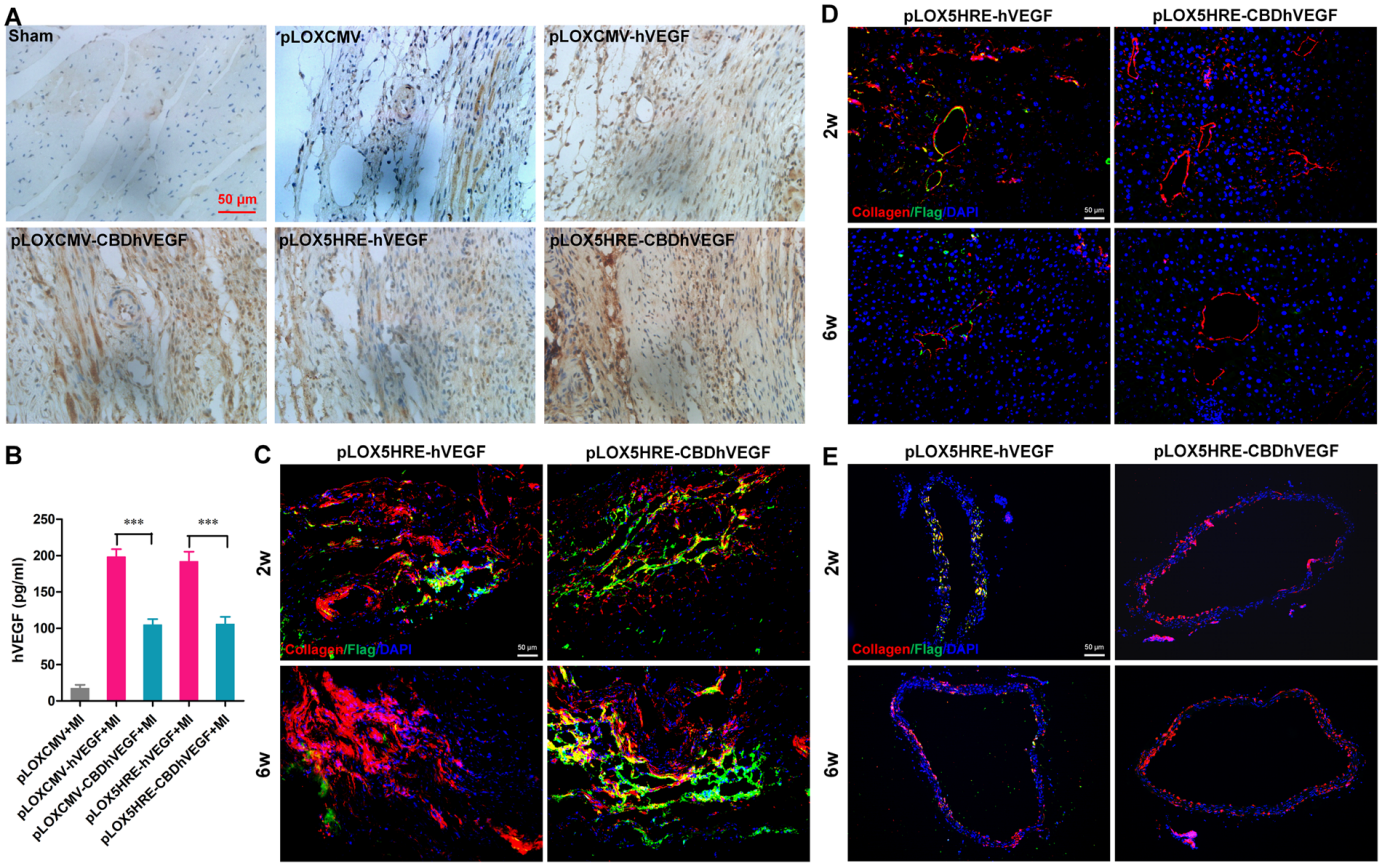
Expression of hVEGF in hypoxic myocardium and serum. (**A**) The expression of hVEGF in hypoxic myocardium was examined by immunohistochemistry using anti-human VEGF-A antibody. Representative images show increasing expression of hVEGF in mice treated with lentivirus expressing hVEGF, especially CBDhVEGF, compared with mice treated with control virus (scale bar = 50 μm). (**B**) Human VEGF production in serum was examined by a human VEGF ELISA assay. Results are presented as mean ± SEM (n = 8), ***p < 0.001. (**C-E**) Co-localization immunofluorescent staining of collagen I (red) and Flag (green) in the infarcted myocardium (**C**), liver (**D**) and descending thoracic aorta (**E**) of mice with injection of pLOX5HRE-hVEGF or pLOX5HRE-CBDhVEGF lentivirus for 2 to 6 weeks.

To further confirm the binding of CBDhVEGF to the hypoxic myocardium, we next examined the expression of Flag tag directly fused with C-terminus of hVEGF by immunofluorescence staining. As shown in Fig. 5A, Flag expression was undetectable in the infarct zone of control group. Moderate expression of Flag was detected in groups injected with lentivirus based on pLOXCMV-hVEGF, pLOXCMV-CBDhVEGF, or pLOX5HRE-hVEGF vectors. However, robust Flag expression was observed in group treated with lentivirus based on pLOX5HRE-CBDhVEGF vector. Moreover, statistic analysis revealed that Flag expression intensity and Flag positive cell (FITC^+^) percentage were significantly higher in CBDhVEGF groups than that in hVEGF groups regardless of CMV or 5HRE promoter, respectively. Interestingly, CBDhVEGF under 5HRE promoter significantly promoted the expression of Flag compared with that under CMV promoter (Fig. 5B and C). We also measured the expression levels of VEGF in infarct zone by immunohistochemistry with anti-VEGF antibody. As shown in Fig. 6A, although moderate production of VEGF was detected in control group, increased productions of VEGF were detectable in experimental groups treated with lentivirus expressing hVEGF or CBDhVEGF. As expected, CBDhVEGF could specifically bind to infarct zone compared with hVEGF alone mediated by lentivirus or with control group. To further confirm the diffusion of hVEGF or CBDhVEGF into peripheral blood, we analyzed the levels of human VEGF in serum. As shown in Fig. 6B, the concentrations of hVEGF in the CBDhVEGF-treated groups were significantly lower than those in hVEGF-treated groups, regardless of CMV or 5HRE promoter.

Next, pLOX5HRE-hVEGF and pLOX5HRE-CBDhVEGF lentivirus were injected into mice with MI treatment for 2 to 6 weeks, tissues including heart, liver and vessel were analyzed to further explore the diffusion of VEGF. As shown in Fig. 6C, more Flag expression (green, reporting VEGF) were detected in the infarcted myocardium in CBDhVEGF group, compared with hVEGF group. This means the possibility that hVEGF may be diffused into peripheral blood. However, there were more Flag signals co-localized with collagen I (red) in liver and vessel (Fig. 6D and E). These findings further demonstrated that hVEGF diffused easily into blood, whereas CBDhVEGF could specifically bind to infarct myocardium. Furthermore, hVEGF, especially CBDhVEGF, could reduce the cell apoptosis levels in the infarcted myocardium, which were confirmed by both cleaved caspase 3 and TUNEL staining (Figure S6).

### Hypoxia-inducible CBDhVEGF promotes angiogenesis after MI

**Figure 7:**
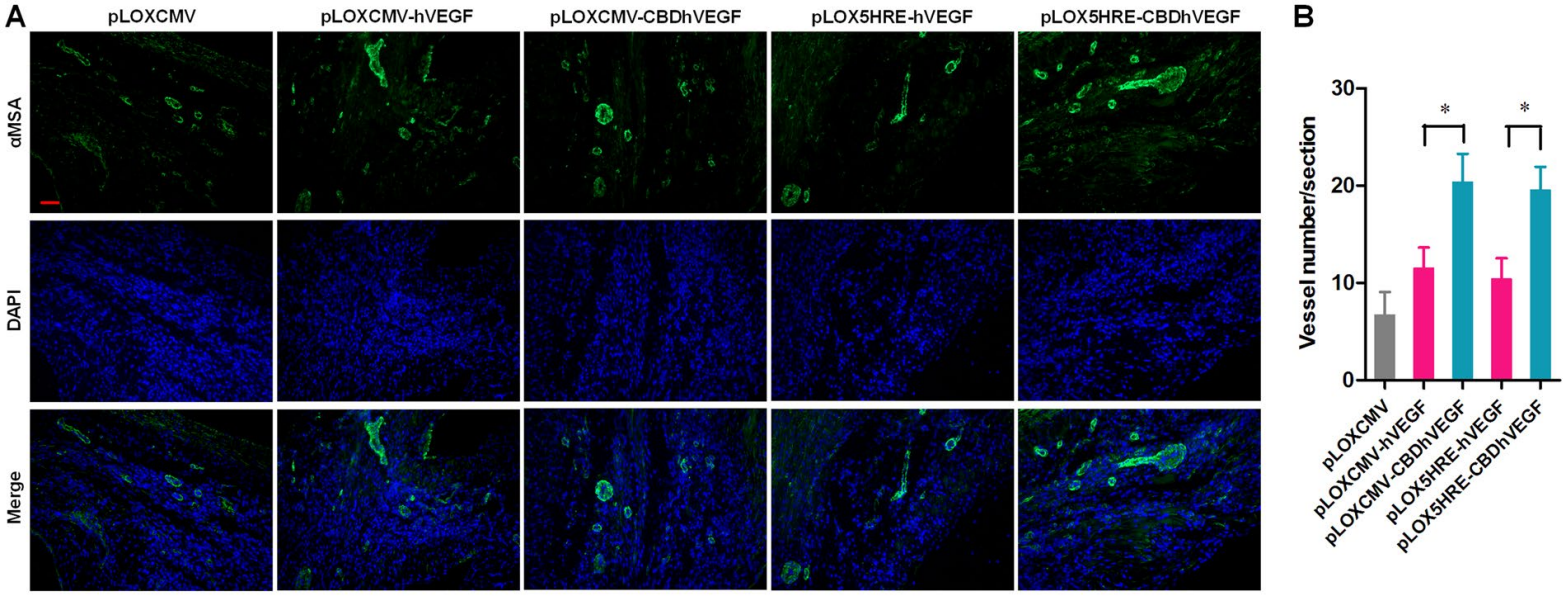
Blood vessel density in the infarct zone. (**A**) Representative pictures of α-SMA (green) and DAPI (blue) stained blood vessels in the infarcted myocardium (scale bar = 50 μm). (**B**) Quantification of blood vessel density in the infarcted myocardium. Results are presented as mean ± SEM (n = 8), **p < 0.01.

To further explore the mechanism by which hypoxia-inducible CBDhVEGF improves cardiac function, the angiogenic efficacy of CBDhVEGF was analyzed by immunofluorescence staining for alpha-smooth muscle actin (α-SMA). As shown in Fig. 7A, only few smaller blood vessels were detected in control group. However, more blood vessels with bigger size and intact structure were observed in experimental groups treated with lentivirus expressing hVEGF or CBDhVEGF. Although hVEGF gene delivery faintly promoted the vessel density compared with control group, there was no significant difference. Whereas, CBDhVEGF gene delivery could significantly increase the vessel density compared with hVEGF, regardless of the CMV or 5HRE promoter (Fig. 7B). Importantly, 5HRE-mediated CBDhVEGF exhibited higher angiogenic capacity like with CBDhVEGF controlled by CMV promoter. Therefore, hypoxia-inducible CBDhVEGF efficiently induced angiogenesis after MI.

## Discussion

In the present study, we found that 5HRE-CBDhVEGF system is hypoxia-responsive and collagen-targeted both *in vitro* and *in vivo*. We demonstrated that the controlled expression of CBDhVEGF mediated by 5HRE promoter specifically binds to ischemic myocardium and efficiently improves cardiac function after MI through promoting angiogenesis in infarct myocardium.

It has been demonstrated that considerable quantity of VEGF diffused into peripheral blood from infarct myocardium after injection of VEGF proteins, thereby reducing the local concentration of VEGF and therapeutic efficiency^7^. Thus, many strategies were focused on localizing and sustaining VEGF proteins at the sites of injury to treat tissue ischemia. Multiple injection is the usual method to maintain the local concentration of VEGF proteins, but this increased the cost and produces adverse effects. Previous studies have revealed that production of type I collagen, the main component of cardiac extracellular matrix, increases in ischemic area after MI^12,13^. Thus, type I collagen may be used as a potential target for the controlled release of growth factors in the infarcted myocardium. Indeed, type I collagen-targeted VEGF and SDF-1α by fusing with collagen-binding domain (CBD) showed great benefits for therapy of MI compared with non-fusion protein alone^5,14^. These previous studies indicated that fusion proteins consist of CBD peptide and growth factors or chemokines may be an efficient strategy to enhance the local concentration of therapeutic factors. However, growth factors including VEGF are rapidly degraded *in vivo* due to their short half-life and this is a major limitation for direct delivery of proteins^16^.

Alternatively, gene transfer technology can provide long-term expression of therapeutic proteins *in vivo* and overcome the shortcomings of short half-life of growth factors. After several decades of endeavour, a growing number of clinical trials of gene therapy successes in both monogenetic and polygenetic conditions have been reported with expected efficiency and safety^21–24^. For gene therapy approaches, lentiviral vectors have been repeatedly shown to have low immunogenicity and ability to transducer primary cells with high efficiency, in addition to carrying a larger therapeutic cargo than other vectors^24–26^. These previous studies suggested that lentiviral vector is a suitable approach for gene therapy. However, uncontrolled overexpression of therapeutic genes may result in serious adverse effects. It has been demonstrated that the unregulated continuous expression of VEGF in myocardium is associated with a high rate of death and formation of endothelial cell-derived intramural vascular tumors^7^. Thus, controlled or regulated expression of VEGF is critical for gene therapy to enhance safety and therapeutic efficiency. Hypoxia is a hallmark of various ischemic diseases such as ischemic heart disease and thus it could be suitable factor for gene therapy to specifically control the expression of VEGF. Hypoxic response elements (HREs) have been reported in the 5′ or 3′ flanking regions of VEGF^27,28^. Previous studies have demonstrated that hypoxia-inducible VEGF based on HREs significantly attenuates infarct size and apoptosis following MI and suggested that HRE is a good candidate for the control of angiogenic gene expression in ischemic myocardium^19,29,30^.

In the present study, we designed a lentiviral vector system expressing CBDhVEGF under control of 5HRE, a hypoxia-responsive promoter. Using this system, we demonstrated that CBDhVEGF expression is controlled by hypoxia both *in vitro* and *in vivo* (Figs 1, 2 and S5), indicating that 5HRE promoter used in this study is hypoxia-responsive as previously reported^17,18^. Thus, 5HRE promoter may be a suitable element for gene therapy for hypoxic disease. Furthermore, CBDhVEGF mediated by 5HRE exhibited similar biological activity to promote endothelial cells viability as hVEGF (Fig. 3A), suggesting that the fusion of CBD has no significant impact on bioactivity of hVEGF. This result is consistent with previous study using proteins injection to treat MI^5^. *In vivo* study demonstrated that pLOX5HRE-CBDhVEGF lentivirus significantly improves cardiac function after MI, compared with pLOX5HRE-hVEGF or control groups (Fig. 4). This beneficial effect could be derived from the increase in CBDhVEGF local concentration in hypoxic myocardium (Fig. 6) as well as the increased blood vessel numbers in infarct zone (Fig. 7). In addition to the capillaries, increased vessel numbers and big vessel were observed in infarct zone following pLOX5HRE-CBDhVEGF lentivirus injection. It is known that bigger vessels are more important for myocardial perfusion because of their greater perfusion capacity, compared with capillaries^31^. In addition, more intact myocardium was also observed in the peri-infarct zone of the pLOX5HRE-CBDhVEGF group than that in pLOX5HRE-hVEGF group, which might be closely related to the increase in cardiomyogenesis or to the decrease in apoptosis resulting from treatment with VEGF^32–34^. As expected, apoptosis of cardiac cells was decreased in pLOX5HRE-CBDhVEGF group compared with pLOX5HRE-hVEGF group (Figure S6). We also constructed lentiviral vectors pLOXCMV-hVEGF and pLOXCMV-CBDhVEGF and compared their therapeutic effects with pLOX5HRE-CBDhVEGF or pLOX5HRE-hVEGF. We found that lentiviral vectors-mediated CBDhVEGF has more therapeutic effects than hVEGF regardless of CMV or 5HRE promoter. In addition, pLOX5HRE-CBDhVEGF exhibited a high therapeutic potential to treat MI like with pLOXCMV-CBDhVEGF. Therefore, pLOX5HRE-CBDhVEGF lentiviral vector could be used as a suitable approach to treat MI due to its high efficiency and controlled expression of VEGF as well as its specific binding to collagen in hypoxic myocardium.

In conclusion, our results demonstrate that lentiviral delivery system for CBDhVEGF driven by 5HRE promoter could significantly improve cardiac function after MI, through enhancing local concentration of VEGF targeting to collagen and promoting angiogenesis in hypoxic myocardium. It is an efficient approach to treat MI, due to its benefits including controlled expression of VEGF by hypoxic condition and specific targeting to collagen in infarcted myocardium. Moreover, hypoxia-inducible 5HRE promoter possesses comparable ability to drive therapeutic gene expression like with the ubiquitous CMV promoter. Therefore, our approach has more extensive applications in gene therapy for hypoxic tissue related diseases by replacing the therapeutic gene. However, additional *in vivo* studies are necessary to evaluate the long-term efficacy and safety of the 5HRE-CBDhVEGF lentiviral vector system for MI treatment.

## Methods

### Animal protocols

C57BL/6 mice at 9–10 weeks of age (20–25 g) were submitted to surgical procedures to induce MI by permanent ligation of the left anterior descending artery (LAD) with a 7–0 prolene suture as previously described^35,36^. All surgical and experimental procedures with mice were approved by the Jinan University Laboratory Animal Committee on Animal Welfare. All animals were treated in accordance with the guidelines of the Laboratory Animal Committee at Jinan University. Immediately after the ligation of LAD, a total of 30 μL of lentivirus (1 × 10^9^ PFU/mL for each) including pLOXCMV, pLOXCMV-hVEGF, pLOXCMV-CBDhVEGF, pLOX5HRE-hVEGF, and pLOX5HRE-CBDhVEGF were injected into 3 sites in the infarct zone through an insulin syringe with an incorporated 29 G needle (BD), respectively. Sham controls were the same as the MI operation but without LAD ligation. Animals were anaesthetized with ketamine (100 mg/kg, i.p.). Animals were analyzed for cardiac function by echocardiography 2 weeks after LAD, and immediately followed by heart isolation for histology analysis (except special mentioned in figure legends, other *in vivo* analyzation was performed 2 weeks after LAD and virus injection).

### Lentiviral vectors design and construction

The pLOX-CMV-MCS (pLOXCMV) and pLOX-CMV-MCS-EF1α-EGFP-Puro (pLOXCMV-E/P) lentiviral vectors were constructed based on the pLOX-TERT-iresTK vector (Addgene, #12245) according to the methods as described previously^37^. The DNA sequence of 5HRE-hCMVmp promoter was synthesized according to 5HRE/GFP plasmid (Addgene, #46926)^18^, and inserted it into the pLOXCMV or pLOXCMV-E/P vectors to replace CMV promoter by *ClaI* and *SpeI* double digestion, the pLOX5HRE and pLOX5HRE-E/P vectors were then constructed.

The ORF of human VEGF-A-Flag fusion fragment without the termination codon was amplified by fusion PCR primer pairs (vegf-F/vegf-R) from the DNA template of pCMV3-VEGFA-Flag plasmid (Sino Biological Inc. Beijing, China, #HG11066-CF), which consists of the human VEGF-A (GenBank No. NM_001171626.1) coding sequence and a Flag tag. A fusion fragment of T2A-mCherry was amplified by the other fusion PCR primer pairs (T2A-mCherry-F/T2A-mCherry-R) from the DNA template of Ngnog-2A-mCherry vector (Addgene, #59995)^38^. To construct the pLOX5HRE-hVEGF-Flag-T2A-mCherry vector (pLOX5HRE-hVEGF), above amplified PCR fragments of hVEGF-Flag and T2A-mCherry were further fused and linked with the linear pLOX5HRE vector digested with *EcoRI* and *XhoI* by the pEASY^®^-Uni Seamless Cloning and Assembly Kit (TransGen Biotech, Beijing, China), according to the manufacturer’s instructions. The vegf-F primer consists of partial sequences from 3′ end of 5HRE promoter and 5′ end of hVEGF-Flag fragment. Both vegf-R and T2A-mCherry-F primers consist of partial sequences from 3′ end of hVEGF-Flag and 5′ end of T2A-mCherry fragments. The T2A-mCherry-R primer consists of partial sequence from 3′ end of T2A-mCherry fragment with stop codon and partial sequence from MCS following *XhoI* restriction site.

We designed a CBD-vegf-F1 primer, containing a CBD coding sequence followed by a linker (GGGGS) and partial sequence from 5′ end of hVEGF, to amplify the CBD-hVEGF-Flag (CBDhVEGF) fragment together with vegf-R primer. The CBDhVEGF fragment was then cloned into pEASY^®^-Blunt Cloning vector (TransGen Biotech, Beijing, China) to construct pEASY-CBDhVEGF vector. A CBD-vegf-F2 primer, containing partial sequence from 3′ end of 5HRE promoter followed by the CBD coding sequence and partial linker sequence, was also designed to amplify the extended CBDhVEGF fragment together with vegf-R primer based on the template of pEASY-CBDhVEGF vector. To constructed pLOX5HRE-CBDhVEGF vector, the extended CBDhVEGF and T2A-mCherry fragments were further fused and inserted into pLOX5HRE vector by the pEASY^®^-Uni Seamless Cloning and Assembly Kit (TransGen Biotech, Beijing, China), as described above.

Both pLOX5HRE-hVEGF and pLOX5HRE-CBDhVEGF vectors were further double digested by *ClaI* and *SpeI* to remove the 5HRE promoters, and replaced them by the full length CMV promoter isolated from pLOXCMV vector by *ClaI* and *SpeI* double digestion. Thereby, pLOXCMV-hVEGF and pLOXCMV-CBDhVEGF vectors were then constructed. Both pLOXCMV-hVEGF and pLOXCMV-CBDhVEGF vectors were further double digested by *EcoRI* and *XhoI*, both hVEGF-Flag-T2A-mCherry and CBD-hVEGF-Flag-2A-mCherry fragments were further isolated and inserted into pLOXCMV-E/P or pLOX5HRE-E/P vector, respectively. Thus, pLOXCMV-hVEGF-Flag-T2A-mCherry-E/P (pLOXCMV-hVEGF-E/P), pLOXCMV-CBD-hVEGF-Flag-T2A-mCherry-E/P (pLOXCMV-CBDhVEGF-E/P), pLOX5HRE-hVEGF-Flag-T2A-mCherry-E/P (pLOX5HRE-hVEGF-E/P), and pLOX5HRE-CBD-hVEGF-Flag-T2A-mCherry-E/P (pLOX5HRE-CBDhVEGF-E/P) were further constructed by normative molecular methods. Moreover, the coding sequence of mCherry was amplified by mCherry-F/mCherry-R primer pairs and subcloned into the MCS of pLOX5HRE-E/P vector by *EcoRI* and *XhoI* double digestion to construct the pLOX5HRE-mCherry-E/P vector. All above vectors have been verified by enzyme digestion and sequencing. The sequences of above PCR primers used in this study are listed in Table S1. The detailed vector design procedure is shown in Fig. S1.

### Cell culture and transient transfection

HEK293T cells were cultured in DMEM medium (Gibco, USA) containing 10% fetal bovine serum (FBS) (Gibco, USA) and 0.5% streptomycin/penicillin solution (10,000 U/ml, Invitrogen), at 37 °C in a 5% CO_2_ incubator. Freezed HEK293T cells were thawed and used for experiments within 10 passages and make sure the individuate experiment was performed using the same patch. For transient transfection experiment, cells were seeded in 24-well plate at a density of 5 × 10^4^ cells/well. After overnight incubation, cells were transfected by pLOX5HRE-mCherry-E/P, pLOXCMV-E/P, pLOXCMV-hVEGF-E/P, pLOXCMV-CBDhVEGF-E/P, pLOX5HRE-hVEGF-E/P, or pLOX5HRE-CBDhVEGF-E/P vectors (0.8 μg for each) using the LipoFiter^TM^ Liposomal Transfection Reagent (Hanbio Biotechnology, Shanghai, China). After transfection for 8 h, cells were further incubated for indicated period in normoxic incubator with 5% CO_2_ or in hypoxic incubator with 5% CO_2_ and 94% N_2_.

### Hypoxia-responsive assay for 5HRE promoter

After transient transfection for 24 or 48 h by pLOX5HRE-mCherry-E/P, pLOXCMV-E/P, pLOXCMV-hVEGF-E/P, pLOXCMV-CBDhVEGF-E/P, pLOX5HRE-hVEGF-E/P, or pLOX5HRE-CBDhVEGF-E/P vectors under normoxic or hypoxic conditions, HEK293T cells were analyzed by laser confocal microscopy to examine the expression of EGFP and mCherry fluorescence reporter genes. Numbers of positive cells with mCherry^+^ and/or EGFP^+^ as well as mCherry fluorescence intensity were calculated by Image-Pro Plus version 6.0 software. The ability of 5HRE promoter was expressed as a percentage of the mCherry^+^ positive cell to the EGFP^+^ positive cells as well as the mCherry fluorescence intensity, respectively. A total of 500 cells from four randomly selected fields were counted. The culture supernatants were analyzed for the released human VEGF by ELISA (R&D Systems, Minneapolis, MN) according to the manufacturer’s instructions. To analyze the hypoxia-responsive ability of 5HRE promoter *in vivo*, lentivirus including pLOXCMV-CBDhVEGF or pLOX5HRE-CBDhVEGF were injected into hearts of mice with or without MI treatment for 6 weeks, heart samples were then analyzed in different time points.

### Biological activities of hVEGF and CBDhVEGF expressed by pLOX5HRE vectors

HEK293T cells were seeded in 6-well plate at a density of 5 × 10^5^ cells/well and transfected by pLOX5HRE-hVEGF or pLOX5HRE-CBDhVEGF vectors, then were incubated under hypoxic conditions for 24 h. The culture supernatants were isolated, analyzed for testing the concentration of hVEGF and CBDhVEGF which were used to treat the type I collagen or the human umbilical vein endothelial cell line (HUVECs) as below. The culture mediums from cells transfected by pLOX5HRE vector were used as the control medium to dilute hVEGF or CBDhVEGF.

HUVECs were purchased from ATCC and cultured with DMEM medium (Gibco, USA) containing 10% fetal bovine serum (FBS) (Gibco, USA), 0.12% Heparin, 1% endothelial cell growth supplement (ECGS) (sigma, USA), and 0.5% streptomycin/penicillin solution (10,000 U/ml, Invitrogen), at 37 °C in a 5% CO_2_ incubator. Ad mentioned above, freezed HUVEC cells were thawed and used for experiments within 10 passages and make sure the individuate experiment was performed using the same patch. HUVECs cell suspension (1 × 10^4^ cells in 100 μL) were seeded into 96-well plates and cultured overnight, the culture medium was then replaced with the medium containing hVEGF or CBDhVEGF with the indicated concentrations from 0 to 2,000 pg/ml. Cell viability of HUVECs was examined by the CC-K8 Cell Counting Kit (Beyotime Biotechnology, China) after incubation for 48 h, according to the manufacturer’s instruction as previously described^39^.

### Collagen-binding assay and biological activity of VEGF after binding to collagen

Type I collagen purchased from Sigma–Aldrich Co. (St. Louis, MO, USA) was added into 96-well plates with the concentration of 3 μg/100 μL per well. The plate was incubated at 4 °C overnight and then dried after discarding the solution. After washing and blocking by 1% BSA in PBS, the plate with 100 μL of serial dilutions of hVEGF or CBDhVEGF per well were incubated for 2 h at 37 °C. After washing three times by PBS, the remaining VEGF binding to type I collagen was examined by the human VEGF ELISA Kit (R&D Systems, Minneapolis, MN). The absorbance values were quantified at 450 nm using a multimode microplate reader (BioTek Instruments, Winooski, VT). The dissociation constants (*K*_d_) of hVEGF and CBDhVEGF from collagen were calculated by the Scatchard analysis. In addition, after treatment with hVEGF or CBDhVEGF at indicated concentration, the collagen-coating plate was further seeded HUVECs at the density of 1 × 10^4^ cells/100 μL. The cell viability was examined by the CC-K8 Cell Counting Kit (Beyotime Biotechnology, China) after incubation for 48 h.

### Lentivirus production

The pLOX-based lentiviruses were prepared in HEK293T cells as previously described^37,40^. In brief, each of lentiviral vectors including pLOXCMV, pLOXCMV-hVEGF, pLOXCMV-CBDhVEGF, pLOX5HRE-hVEGF, and pLOX5HRE-CBDhVEGF, was co-transfected into HEK293T cells together with the packaging plasmids pCMVR8.74 (Addgene, #22036) and pMD2.G (Addgene, 12259) using the LipoFiter^TM^ Liposomal Transfection Reagent (Hanbio Biotechnology, Shanghai, China), thereby generating viruses. The viral supernatant was concentrated with the Lenti Virus Concentration Reagent (Biomiga, CA, USA) and titrated in HEK293T cells. High titre virus (1 × 10^9^ PFU/mL) was re-suspended in PBS. A large number of small stock aliquots (10 μL) were made and frozen at −80 °C for myocardium injection in mouse.

### Echocardiography

Two weeks after the ligation of LAD, transthoracic echocardiography was performed using the Vevo® 2100 ultrasound system (Visualsonics, Toronto, Canada) equipped with a high-frequency (30 MHz) linear array transducer. Parasternal long-axis, short-axis, and two apical four-chamber views were used to obtain 2-dimensional and M-mode images. The left ventricular (LV) end-systolic dimension (LVDs) and end-diastolic dimension (LVDd) were measured in M-mode tracings at the mid-papillary level. The LV end-diastolic volume (LVEDV), the LV end-systolic volume (LVESV), the LV stroke volume (LVSV) and the LV cardiac output (LVCO) were measured to evaluate cardiac function. The ejection fraction (EF) and fractional shortening (FS) were calculated by the Vevo® 2100 ultrasound system automatically.

### Serum hVEGF and CBDhVEGF Levels

After echocardiography analysis, the serum was collected and measured with a human VEGF ELISA Kit (R&D Systems, Minneapolis, MN) to evaluate the leakage of hVEGF and CBDhVEGF expressed in infarct zone into blood.

### Histological Analysis

Following serum collection, hearts (or livers and descending thoracic aortas for double staining by collagen I and Flag) were excised rapidly and transected into two segments through the infarct zone. One segment was fixed in 4% paraformaldehyde for 48 h and embedded in paraffin. The other segment was embedded in Tissue-Tek optimal cutting temperature compound (OCT) (Sakura, USA) for frozen section. Paraffin sections (5 μm) were prepared and stained with hematoxylin and eosin (H&E) or with Masson trichrome for morphological and fibrosis analysis, respectively. Moreover, immunohistological staining was performed on the paraffin sections with anti-VEGF-A antibody (5 μg/mL, Abcam, USA) to evaluate the retained VEGF in infarct zone. Cell apoptosis in infarct zone was evaluated by using *In Situ* Cell Death Detection Kit, POD (Roche) according to the manufacturer’s instruction. Frozen sections (4 μm) were incubated with the primary antibodies against Flag (20 μg/mL, Sigma-Aldrich, USA), against CD31 (1:500 dilution, Abcam), against collagen I (1:200 dilution, Abcam), against alpha-smooth muscle actin (α-SMA) (1:100 dilution, Abcam, USA), or against cleaved caspase3 (1: 400 dilution, Cell Signaling, USA) at 4 °C overnight. After washing, the sections were incubated with the FITC-conjugated goat anti-mouse or goat anti-rabbit IgG (1:500 dilution, Abcam) at room temperature for 2 h, respectively. The nuclei were counterstained with DAPI. Flag signaling was used to evaluate the expression of hVEGF-Flag fusion protein. The α-SMA signaling was used to evaluate the density of the capillary vessels. In addition, mCherry expression was directly examined using frozen section to examine the hypoxic responsiveness of 5HRE promoter *in vivo*. For statistic analysis, one representative section from each mouse was collected to calculate average values.

### Statistic analysis

All data are presented as the mean ± SEM. Statistical analysis was performed using one-way ANOVA followed by Dunnett’s multiple comparison tests. Comparisons between two groups were analyzed using unpaired Student’s *t*-test. A *p* value of less than 0.05 was considered to be statistically significant.

## Electronic supplementary material

Supplementary information
