# Supplementary material for: Gene delivery of hypoxia-inducible VEGF targeting collagen effectively improves cardiac function after myocardial infarction
Source: Sci Rep. 2017 Oct 16;7:13273. doi: 10.1038/s41598-017-13547-1 (PMC5643404; doi:10.1038/s41598-017-13547-1)
Supplement: Supplementary file 1 — Supplementary information [file 41598_2017_13547_MOESM1_ESM.pdf]

Gene delivery of hypoxia-inducible VEGF targeting collagen effectively improves cardiac function after myocardial infarction

Jing-Bo Xia [1#], Hai-Yan Wu [1#], Bing-Lin Lai [1#], Li Zheng [2#], Deng-Cheng Zhou [1], Zao-Shang Chang [1], Cheng-Zhou Mao [1], Guang-Hui Liu [1], Kyu-Sang Park [3], Hui Zhao [4], Soo-Ki Kim [5], Guo-Hua Song [6]*, Dong-Qing Cai [1]*, Xu-Feng Qi [1]*

[1] *Key Laboratory of Regenerative Medicine of Ministry of Education, Department of Developmental & Regenerative Biology, Jinan University, Guangzhou 510632, China*

[2] *College of Environmental Science and Engineering, Guangdong University of Technology, Guangzhou 510006, China.*

[3] *Department of Physiology, Wonju College of Medicine, Yonsei University, Wonju, Gangwon 220-701, Korea*

[4] *Key Laboratory of Regenerative Medicine of Ministry of Education, School of Biomedical Sciences, Faculty of Medicine, The Chinese University of Hong Kong, Hong Kong SAR, China*

[5] *Department of Microbiology, Yonsei University Wonju College of Medicine, Wonju, Gangwon 220-701, Korea*

[6] *Institute of Atherosclerosis, TaiShan Medical University, Tai'an 271000, China*

[#] These authors contributed equally to this work and should be considered first authors.

***Correspondence:** Key Laboratory of Regenerative Medicine, Ministry of Education, Jinan University, Guangzhou 510632, China. Tel: +86-20-85222687; Fax: +86-20-85222711. E-mail: qixufeng@jnu.edu.cn (Xu-Feng QI), tdongbme@jnu.edu.cn (Dong-Qing Cai) or girl_sapphire@hotmail.com (Guo-Hua Song).

Supplementary information

Fig. S1

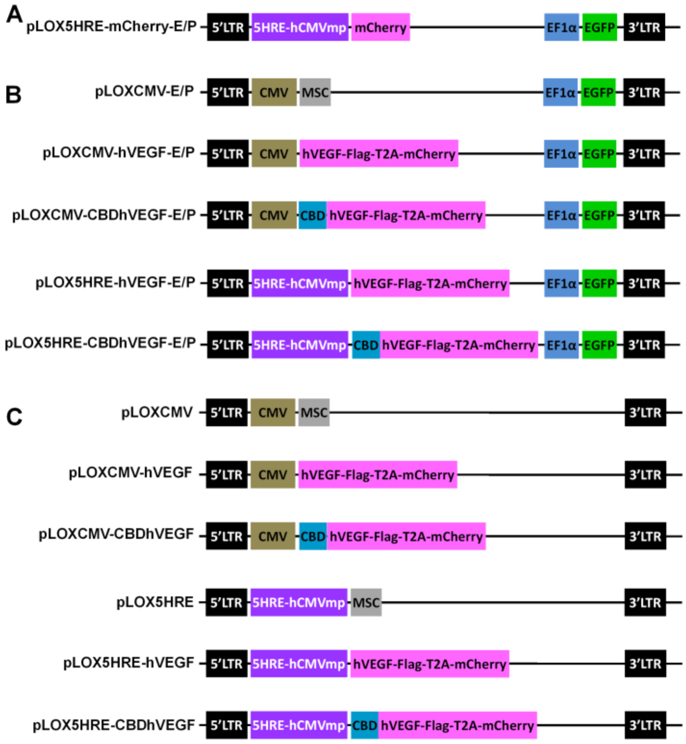

Figure S1. Schematic drawing of the constructs of lentiviral vectors used in this study. (A) Construct of pLOX5HRE-mCherry-E/P vector. (B) Constructs of lentiviral vectors based on pLOXCMV-E/P backbone. (C) Constructs of lentiviral vectors based on the pLOXCMV backbone.

Fig. S2

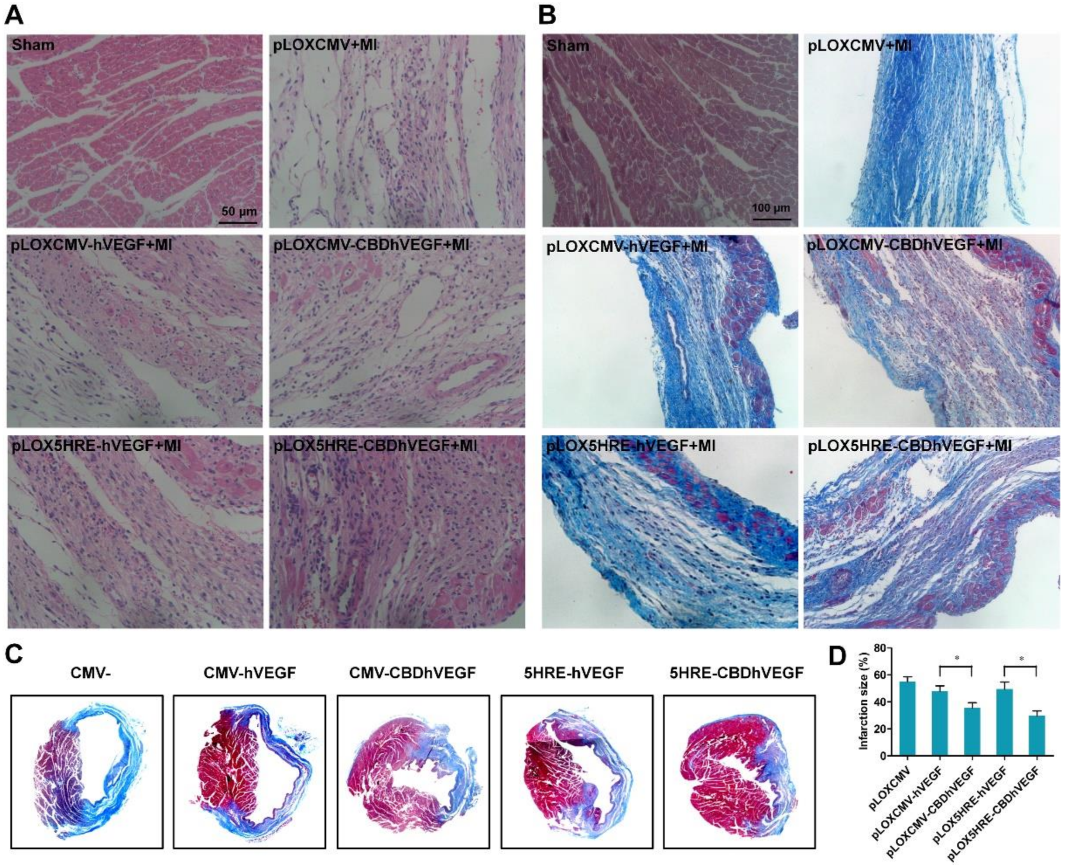

Figure S2. Histological analysis of infarcted myocardium. (A) Heart sections were stained with hematoxylin and eosin (H&E) showing intact myocardiocyte morphology in infarct zone of mice treated with lentivirus expressing hVEGF or CBDhVEGF compared with mice treated with control virus. (B and C) Heart sections were stained with Masson trichrome. Representative images of infarct zone (B) and whole left ventricle (C) showed the reduced fibrosis of myocardium in mice treated with hVEGF-expressed lentivirus, especially in mice treated with CBDhVEGF-expressed lentivirus, compared with control. (D) The proportion of scar tissues (blue) in the left ventricle was determined by calculating the ratio of the area of scar to the left ventricular area (n =5), *p<0.05.

Fig. S3

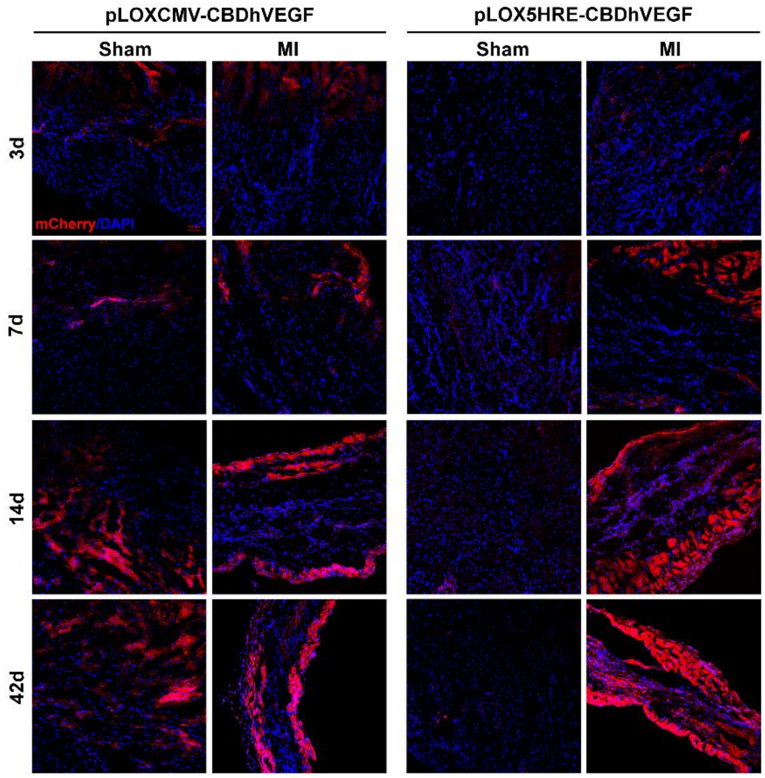

Figure S3. ***In vivo*** **hypoxia-responsive ability of 5HRE promoter.** Lentivirus including pLOXCMV-CBDhVEGF or pLOX5HRE-CBDhVEGF were injected into mice with or without MI treatment for 6 weeks, report gene (mCherry, red) expression in cardiac area with injection was examined by fluorescence microscopy at 3, 7, 14 and 42 days. Representative images showed 5HRE promoter effectively drives target gene expression in the infarcted myocardium area, but not in sham-operated heart. However, CMV promoter drives target gene expression both in MI and sham-operated hearts.

Fig. S4

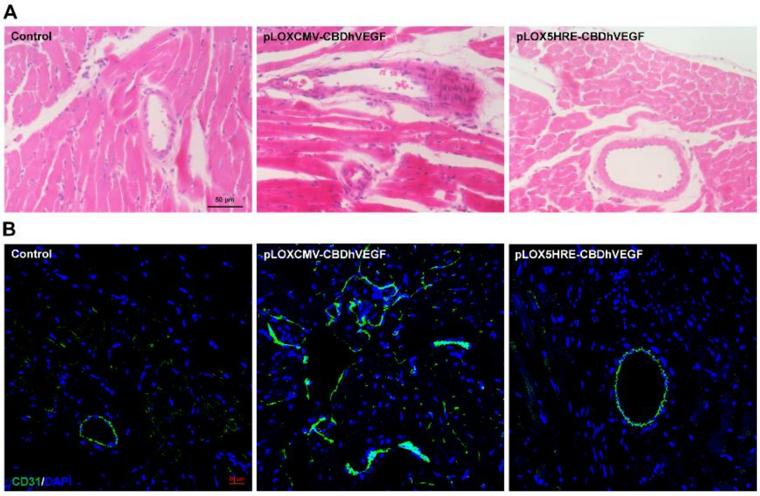

Figure S4. Continuous expression of VEGF driven by CMV promoter induces abnormal angiogenesis. Sham-operated mice were injected by pLOXCMV-CBDhVEGF or pLOX5HRE-CBDhVEGF lentivirus for 6 weeks, heart tissue was analyzed by H&E and immunofluorescence staining. (A) Representative images of H&E staining showed abnormal angiogenesis in pLOXCMV-CBDhVEGF group, compared with pLOX5HRE-CBDhVEGF group and control group. (B) Representative images of immunofluorescence staining with anti-CD31 antibody indicated that pLOX5HRE-CBDhVEGF group has normal vessels like that in control group. However, abnormal angiogenesis was detected in pLOXCMV-CBDhVEGF group.

Fig. S5

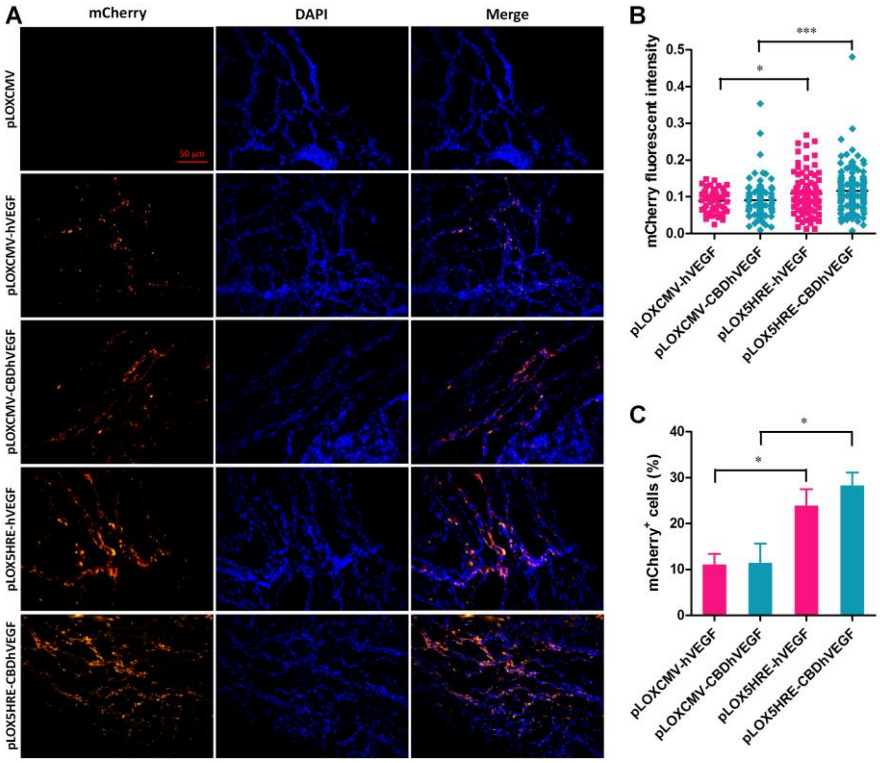

Figure S5. *In vivo* hypoxia-responsive ability of 5HRE-hCMVmp promoter. The expression of mCherry reporter gene, which was fused with VEGF via a T2A peptide, in ischemic myocardium was directly detected by fluorescence microscopy. (A) A representative microscopic image for each condition is shown (scale bar=50 μm). (B) The quantification of mCherry fluorescence intensity was performed as described in Methods section. Results are presented as mean ± SEM (n=8), *$p<0.05$, ***$p<0.001$. (C) Percentage ratio of mCherry positive cells was examined as described in Methods section. Results are presented as mean ± SEM (n=8), *$p<0.05$.

Fig. S6

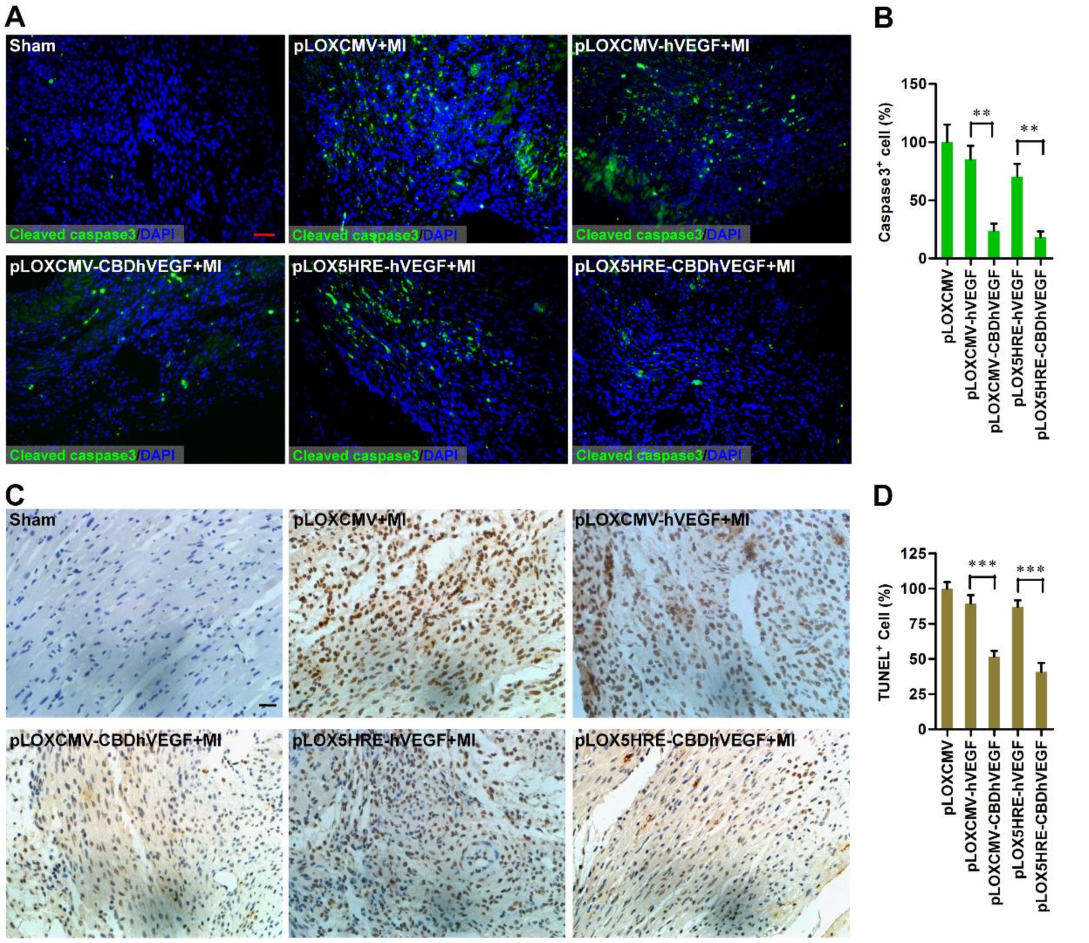

Figure S6. Cell apoptosis analysis in hypoxic myocardium. (A and B) Heart sections were subjected to immunofluorescence staining with anti-cleaved caspase 3 antibogy. (A) Representative images show decreasing cell apoptosis in infarct zone of mice treated with hVEGF, especially CBDhVEGF-expressed lentivirus, compared with mice treated with control virus (scale bar=50 μm). (B) Apoptosis level was evaluated by the relative ratio of Caspase3-positive cells. The level of caspase3-positive cells in pLOXCMV group was set to 100%. Results are presented as mean ± SEM (n=8), **p<0.01. (C and D) Heart sections were subjected to *in situ* cell death detection by TUNEL staining, and suggested reduced cell apoptosis in mice treated with hVEGF- or CBDhVEGF-expressed lentivirus compared with control group (scale bar=20 μm). (C) Representative images are shown in infarct zone. (D) Apoptosis level was evaluated by the relative ratio of TUNEL-positive cells. The level of TUNEL-positive cells in pLOXCMV group was set to 100%. Results are presented as mean ± SEM (n=8), ***p<0.001.

Supplementary Table

Table S1. Fusion PCR primer pairs used in this study

PCR product	Primers	Sequences (5’----3’)
hVEGF	vegf-F	agatctctagaactagtgatcagaattcgccaccatgaactttctgctgtcttgggtgcatt
	vegf-R	gacatcccctgcttgtttcaacagggagaagttagtggccttatcgtcgtcatccttgtaatcagagcct
T2A-mCherry	T2A-mCherry-F	atgacgacgataaggccactaacttctccctgttgaaacaagcaggggatgtc
	T2A-mCherry-R	gcagatccttcggatccgtcgactcgagctacttgtacagctcgtccatgccg
CBDhVEGF	CBD-vegf-F1	gccaccatgaccaagaagactctgagaaccggtggaggcggtagcaactttctgctgtcttgggtgcatt
	CBD-vegf-F2	agatctctagaactagtgatcagaattcgccaccatgaccaagaagactctgagaaccggtggaggcggt
